# Warner’s Pocket Medical Dictionary of Today; over 10,400 Definitions

**Published:** 1899-09

**Authors:** 


### Books and Magazines.

WARNER'S POCKET MEDICAL DICTIONARY OF TODAY; OVER 10,400 DEFINITIONS.—Complete tables of arteries, muscles, nerves, bacteria, bacilli, spirilli, streptococci, etc. Also an accurate posological table. 413 pages. 304 pages of definitions. Table of arteries, 6 pages. Table of muscles, 23 pages. Table of bacilli, bacteria, micrococci, 10 pages. Table of nerves, 13 pages. Table of spirilli and streptococci, 2 pages. A most complete dose table occupying 16 pages. Especially recommended to students for quick reference. The tables are excellently arranged for memorizing nerves, arteries, muscles, etc. Warner's Pocket Medical Dictionary of today is more than a dictionary. Its valuable tables make it a general book of reference. Bound in flexible leather. Easily carried in the pocket. Round corners. Second edition just off press. The definitions comprise 10,400 terms and words used in medicine and the allied sciences. The latest coined words such as Radiograph, X-rays, Roentgen rays, etc., are all in new edition. It is "up-to-date" in every particular. New type. Excellent paper. Easily read. Definitions are clear, concise and comprehensive. Busy doctors who wish to look up a word or its definition and look it up quickly will find this dictionary a handy book of reference. Price 75 cents. Remit stamps or money order. Wm. R. Warner & Co., 639 North Broad street, Philadelphia. 52 Maiden Lane, New York. 197 Randolph street, Chicago.

